# Retraction: Multidrug-Resistance Related Long Non-Coding RNA Expression Profile Analysis of Gastric Cancer

**DOI:** 10.1371/journal.pone.0226210

**Published:** 2019-12-03

**Authors:** The PLOS ONE Editors

Following publication of this article [1], the authors raised to the attention of the *PLOS ONE* Editors that Fig 5Aa showed an incorrect β-tubulin loading control panel. Further concerns were raised as follows:

- In Fig5Aa, the β-tubulin panel is similar to lanes 2–4 of the β-tubulin panel in Fig 5Ba.
- In Fig 5Ba, lanes 1 and 2 of the MDR1 panel are similar to lanes 3 and 2 of the IFIT2 panel of Fig 5Aa (flipped horizontally) in [2].
- In Fig 5Ba, the β-tubulin panel is similar to the tubulin panel of Fig 5Ba in [2].
- In Fig 5Ba, lanes 2–4 of the β-tubulin panel are similar to the tubulin panel of Fig 4Fb in [2].
- In Fig 5Ba, lane 1 of the β-tubulin panel is similar to lane 3 of the tubulin panel of Fig 4Fa in [2].
- In Fig 5Ca, there is a vertical discontinuity between lanes 2 and 3 of the ARNT panel.

The authors have repeated the western blot experiments in support of the original claims; however, the underlying blots for Fig 5 and the datasets underlying the charts in Figs 5 and 6 are not available.

The original western blot image files are not available, and thus it was not possible to resolve the concerns regarding the images in the published Fig 5. As these concerns question the integrity and reliability of the reported results, the *PLOS ONE* Editors retract this article.

Daiming Fan agreed with the retraction. YW, KW, ZY, QZ, Dongmei Fan, PX, YN either did not respond directly to the retraction decision or could not be reached.

## References

[1] Wang Y, Wu K, Yang Z, Zhao Q, Fan D, Xu P, et al (2015) Multidrug-Resistance Related Long Non-Coding RNA Expression Profile Analysis of Gastric Cancer. PLoS ONE 10(8): e0135461 10.1371/journal.pone.0135461 26291830 PMC4546299

[2] Feng X, Wang Y, Ma Z, Yang R, Kiang S, Zhang M, et al (2014) MicroRNA-645, up-regulated in human adencarcinoma of gastric esophageal junction, inhibits apoptosis by targeting tumor suppressor IFIT2. BMC Cancer 14: 633 10.1186/1471-2407-14-633 25174799 PMC4161885

